# Albinism: epidemiology, genetics, cutaneous characterization, psychosocial factors^☆^^☆☆^

**DOI:** 10.1016/j.abd.2019.09.023

**Published:** 2019-09-30

**Authors:** Carolina Reato Marçon, Marcus Maia

**Affiliations:** a Pro-Albino Program Dermatology Clinic, Department of Medicine, Santa Casa de Misericórdia de São Paulo, São Paulo, SP, Brazil; b Department of Oncology Dermatology Clinic, School of Medical Sciences, Santa Casa de Misericórdia de São Paulo, São Paulo, SP, Brazil

**Keywords:** Albinism, Albinism, oculocutaneous, Keratosis, actinic, Sunscreening agents, Skin neoplasms, Social stigma

## Abstract

Oculocutaneous albinism is an autosomal recessive disease caused by the complete absence or decrease of melanin biosynthesis in melanocytes. Due to the reduction or absence of melanin, albinos are highly susceptible to the harmful effects of ultraviolet radiation and are at increased risk of actinic damage and skin cancer. In Brazil, as in other parts of the world, albinism remains a little known disorder, both in relation to epidemiological data and to phenotypic and genotypic variation. In several regions of the country, individuals with albinism have no access to resources or specialized medical care, and are often neglected and deprived of social inclusion. Brazil is a tropical country, with a high incidence of solar radiation during the year nationwide. Consequently, actinic damage and skin cancer occur early and have a high incidence in this population, often leading to premature death. Skin monitoring of these patients and immediate therapeutic interventions have a positive impact in reducing the morbidity and mortality associated with this condition. Health education is important to inform albinos and their families, the general population, educators, medical professionals, and public agencies about the particularities of this genetic condition. The aim of this article is to present a review of the epidemiological, clinical, genetic, and psychosocial characteristics of albinism, with a focus in skin changes caused by this rare pigmentation disorder.

## Introduction

### Etiology, definition, and clinical condition

**Figure 1:**
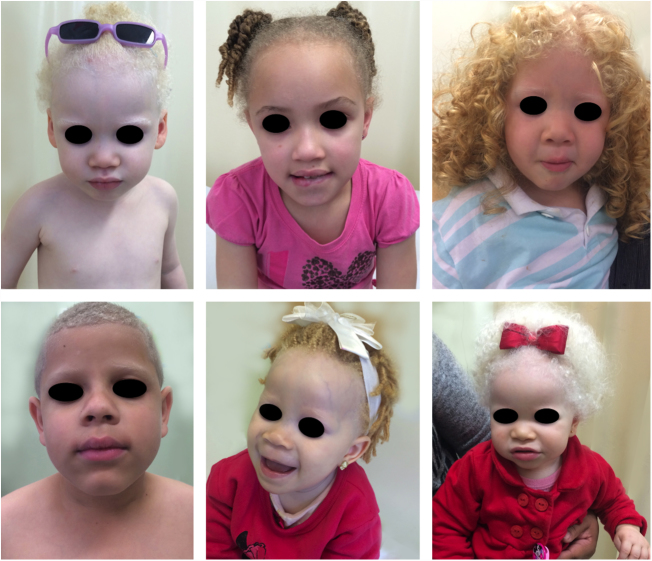
Phenotype in albinism. Wide phenotypic variability among children with albinism.

**Figure 2:**
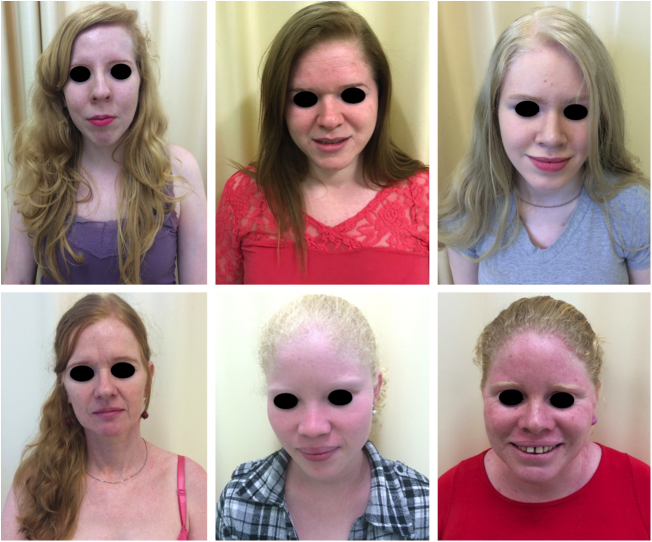
Phenotype in albinism. Wide phenotypic variability among women with albinism.

**Figure 3:**
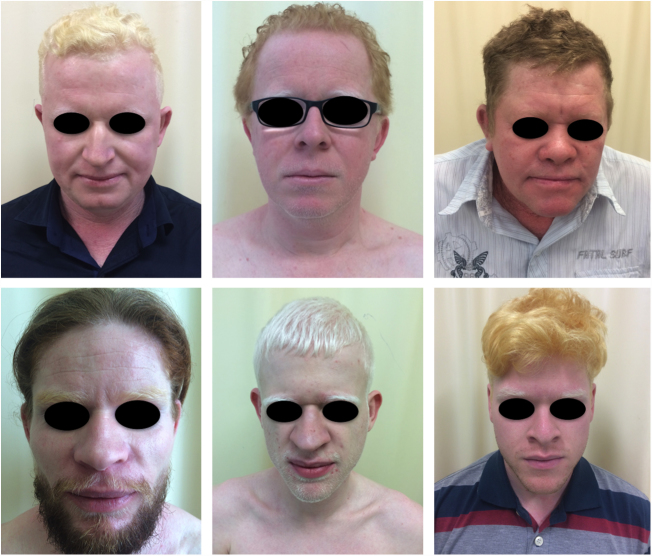
Phenotype in albinism. Wide phenotype variability among men with albinism.

Oculocutaneous albinism (OCA) is an autosomal recessive disorder caused by the complete absence or reduction of biosynthesis of melanin in melanocytes. Patients with albinism have a normal number of melanocytes in the epidermis and follicles, but the melanin pigment is totally or partially absent.^1^, ^2^, ^3^, ^4^ Individuals with oculocutaneous albinism are unable to oxidize tyrosine into dopa through tyrosinase. This inability to produce pigment causes pale complexion, white or fair hair, and red eyes, as light reflects blood vessels in the retina, or greenish-blue or light brown eyes, if there is pigment formation in the iris. In fact, the phenotypic variability of albinism is broad, ranging from complete absence of pigmentation of the hair, skin, and eyes to mild depigmentation (Figure 1, Figure 2, Figure 3).^5^, ^6^, ^7^

Due to the reduction or absence of melanin, albinos are highly susceptible to the harmful effects of ultraviolet (UV) radiation and are at greater risk of actinic damage.^5^, ^8^, ^9^ Clinical management should include education of albinos and family members on the importance of preventing sun exposure and about methods to protect against UV radiation.

Many albinos develop actinic keratosis or skin cancers before reaching the age of 30 years.^5^, ^8^, ^9^, ^10^, ^11^, ^12^ The sequelae of skin cancer are among the leading causes of early death in albino patients.^11^, ^12^ Skin cancer occurs in young adults among albino patients with non-photoprotected exposure to sun. In those patients, skin cancer is invariably multiple and biologically aggressive in nature, although melanoma is rare.^5^, ^8^, ^9^ The cancer typically occurs on the head or neck, areas usually more exposed to solar radiation. Given their high sensitivity to UV light, albinos need total sun protection and should undergo regular skin exams every six months or less.^5^, ^8^, ^9^

Reduced visual acuity, refractive errors, iris translucency, nystagmus, foveal hypoplasia, fundus hypopigmentation, and abnormal decussation of optic nerve fibers at the chiasm are also common features in albinos. This misrouting is characterized by excessive crossing of fibers at the optic chiasm, which can result in strabismus and reduced stereoscopic vision. In addition, photophobia may be severe. Albinos are also ocularly more susceptible to the harmful effects of UV radiation^5^, ^6^, ^7^ and most individuals with albinism have some degree of low visual acuity.

## Albinism types and epidemiology

Albinism can occur in syndromic and non-syndromic forms. In syndromic forms of albinism such as the Hermansky-Pudlak and Chediak-Higashi subtypes, hypopigmentation and visual impairments coexist with more severe pathological abnormalities. Hermansky-Pudlak syndrome can present with immunological changes, interstitial pulmonary fibrosis, granulomatous colitis, and hemorrhagic diathesis secondary to platelet alterations. Chediak-Higashi syndrome, besides hypopigmentation, can manifest with hematologic changes, high susceptibility to infections, bleeding, and neurological problems.^13^, ^14^, ^15^, ^16^, ^17^ Albinism can also be expressed by the exclusively ocular form (OA1 and FHONDA syndrome).^5^, ^18^, ^19^

To date, 19 genes have been linked to the different clinical presentations of albinism, including seven for OCA. Four main types of non-syndromic albinism were initially described: from OCA Type 1 (A and B) to OCA4. The OCA1A type is the most severe, with a total absence of melanin production throughout life, whereas the other forms – OCA1B, OCA2, OCA3, and OCA4 – exhibit some accumulation of pigments over time. Mutations in the TYR, OCA2, TYRP1, and SLC45A2 genes are the main cause of oculocutaneous albinism.^2^, ^5^, ^10^ Recently, another two new genes, SLC24A5 and C10orf11,^20^ have been identified as responsible for causing OCA6 and OCA7, respectively, giving a total of seven different types of albinism. A locus was also mapped in the region of the human chromosome 4q24, the genetic cause of OCA5.^21^

However, there remain a substantial number of albinism cases without molecular identification, suggesting that more genes are associated with the condition. In addition, the lack of knowledge of the underlying mechanisms by which a genetic mutation induces a deleterious functional effect on the gene's product implies that the disorder cannot yet be fully understood.^21^

In Brazil, albinism is clinically diagnosed based on the presence of typical cutaneous abnormalities and ocular findings. The distinction between albinism subtypes based on clinical characteristics and the broad phenotypic heterogeneity of the disorder hinders the establishment of phenotypic and genetic correlations, and there is extensive overlapping of different forms of the disease. Molecular studies to define the exact type of mutation are therefore necessary. However, this test is currently not available under the Brazilian public health system.^2^, ^3^, ^4^

Albinism is a genetic disorder that affects individuals of all social classes and countries worldwide, albeit at different prevalence rates. The global incidence of albinism is 1:20,000 individuals, with a lower rate in the United States (1:37,000),^1^, ^7^, ^22^ while the highest rate reported in the literature to date is amongst the Cuna indigenous people (in Panama and Colombia), who have an estimated incidence of 6.3 per 1000 population.^23^ High rates have also been reported in Africa.^22^, ^23^ In Tanzania, Luande et al.^24^ estimated there were 700 albinos living in Dar es Salaam, representing a prevalence of 1:1500. In sub-Saharan Africa, 1:5000 to 1:15,000 are affected by albinism.^25^ A review study published in 2006 revealed that seven publications contained epidemiological data on the prevalence of albinism in South Africa, Zimbabwe, Tanzania, and Nigeria.^26^ The prevalence of albinism in these studies ranged from 1:15,000 in the mid-eastern state of Nigeria^27^ to 1:1000 among the Tonga tribe of Zimbabwe, an isolated rural community.^28^ Albinism is considered a relatively common hereditary condition among populations of South Africa. Besides the limited geographical mobility, consanguinity, together with other traditional marriage practices, may also be pertinent factors in assessments of current and future trends of albinism prevalence.^22^, ^27^, ^28^, ^29^

The frequency of different types of OCA differs according to the population. OCA1 is the most commonly found subtype in Caucasians, accounting for 50% of all cases worldwide.^30^, ^31^ OCA2, or brown OCA, is responsible for 30% of cases worldwide and is more common in Africa, where it affects an estimated 1:10,000 and more than 1:1000 among certain populations.^27^, ^32^ This is largely due to a highly frequent deletion of OCA2 found in the African population.^32^, ^33^, ^34^, ^35^ OCA3, or red OCA, is practically nonexistent in Caucasians, but affects approximately 1:8500 individuals in South Africa, or 3% of all cases globally.^31^ OCA4 is also rare in Caucasians, and likewise among Africans, but accounts for 17% of all cases worldwide and, in Japan, is diagnosed in one out of every four people affected by OCA. In Japan and China, the predominant form is OCA1, followed by OCA4.^31^, ^36^ New genes and mutation are being discovered around the world, such as that responsible for OCA5 identified in Pakistan,^37^ that for OCA6, first identified in China, but now reported in other populations,^38^, ^39^ and that for OCA7, identified in a family from the Faroe islands and in Denmark.^20^ The Hermansky-Pudlak syndromic form has a high incidence in the Puerto Rican population^40^, ^41^ and is relatively prevalent in some populations: for example, a recent European prospective study assessing the clinical and genetic characteristics of a group of patients (33 children and 31 adults) seen at a specialized day hospital revealed a prevalence of 7.8%.^42^ Chediak-Higashi syndrome is universally rare and is described in some European regions and Asia.^16^, ^17^

In Brazil, the epidemiology of albinism has not been mapped. There are scant epidemiological studies and no information is held in government databases (the census of the Brazilian Institute of Geography and Statistics [IBGE], or the National Health System Database [DATASUS]) on the incidence of the genetic disorder in the country. The incidence is thought to be higher in regions with a greater prevalence African descent, such as the Northeast. The population of Bahia, Brazil's third most populous state, is mostly of African or mixed descent. Owing to the high presence of African ancestry and the fact the region was the point of entry for African slaves during the colonial period, Bahia is believed to have the highest incidence of albinos in the country.^43^, ^44^ In a study on the profile of albinism in the state of Bahia, 70% of the albinos declared they were of African or mixed ethnicity.^44^ Another study conducted in the city of Salvador (capital of Bahia) revealed cases of albinism in 44% of the 163 districts and locations of Salvador, where 17% of places had a prevalence of more than 1:10,000 and 8.5% of over 2:10,000. The districts with higher albinism rates had a high proportion of African descent. The proportion of albinos in one of the regions investigated, called Ilha de Maré, whose population descended from Quilombos (former escaped-slave communities), exceeded 1:1000.^43^

Another area of Brazil studied for its high incidence of albinism is the city of Lençóis, in the north of Maranhão state. Given its small population and remote location, consanguinity is high and the supposed rate of albinism in the 1970s and 1980s was considered one of the highest in the world. The region now has fewer albinos, since many migrated to other regions and/or met early deaths due to skin cancer (according non-published reports). There are also isolated reports of albinism in many indigenous communities of Brazil (in states of Pará, Acre, Paraná, São Paulo, Mato Grosso; data reported from the internet).^45^, ^46^, ^47^

The few studies and reports available in the scientific literature suggest that figures for albinism in Brazil resemble those of Africa. However, it is unclear whether these numbers reflect the true situation in the country, highlighting the need for the government to register albinism in official databases or conduct population-based studies that provide a reliable estimate of these figures. Thus, there is a general paucity of studies mapping the epidemiology of albinism in Brazil. Further studies are needed to provide a deeper understanding on the distribution and incidence of this genetic disorder in the country and the consequent devising of more objective and assertive strategies for the condition.

The occurrence of albinism is associated with difficulties and disadvantages, resulting from the genetic disorder and social segregation. There is stigma related to the disease that affects albinos and their families. Reports of studies carried out in Bahia, data from the Association of Persons Living with Albinism in Bahia* State [APALBA] (*http://apalba-albinosdabahia.blogspot.com), informal global references, and the practical experience of the authors indicate that albinos in Brazil, even in major urban centers, suffer prejudice and social exclusion, as well as limited access to specialized medical healthcare and resources. These factors contribute to an increase in the morbimortality associated with the condition, including actinic damage and skin cancer.^44^, ^45^, ^46^, ^47^, ^48^

## Physiopathology – albinism, actinic damage, and skin cancer

### Physiopathology of melanin biosynthesis and oculocutaneous albinism

Skin pigmentation varies among individuals and is determined by multiple factors, including the number and metabolic activity of melanocytes in the base layer of the epidermis, the melanogenic activity of melanosomes within these melanocytes, and variations in the number, size, and distribution of melanosomes. Differences in types of melanin, the degree of branching of the dendritic protrusions of melanocytes, and in the transport of melanosomes from these protrusions to keratinocytes also affect skin pigmentation.^49^, ^50^

Melanocytes have an ectodermic origin in the neural crest, evolving with cutaneous (hair, skin) or extracutaneous (eyes, cochlea, leptomeninges) migration. Some genes control the proliferation and differentiation of cells from the neural crest and regulate the migration of precursor melanocytes to their final positions. The microphthalmia transcription factor (MITF) is the master regulator of development, function, and survival of melanocytes^51^ and is responsible for modulating the expression of some specific proteins in these cells.^52^ After differentiation of melanocytes, MITF regulates the expression of genes during exposure to ultraviolet radiation (UVR), promoting tanning of the skin.^53^

Melanin is a polymer pigment produced in melanocytes. Its synthesis occurs through enzyme reactions, which convert tyrosine into melanin through the tyrosinase enzyme. Melanin biosynthesis is regulated by a number of factors, particularly the melanocortin-1 receptor (MC1R) in melanocytes and its ligand, the alpha-melanocyte stimulating hormone (α-MSH). Cytokines and growth factors from the environment and the degree of basal activity of tyrosinase, of tyrosinase-related protein 1 (TRP1), and of membrane-associated transporter proteins are additional factors regulating this biosynthesis.^50^, ^51^, ^52^, ^53^, ^54^

One of the important functions of melanin is to protect the skin and eyes from the harmful effects of UV. The melanin produced through this modulation is transferred to adjacent keratinocytes, where a covering around the nucleus is thus formed, protecting the DNA from damage induced by UV radiation.^50^, ^55^ The degree of skin pigmentation is inversely correlated with risk of sun-induced skin cancer.^53^

### Genetic mutations involved in albinism

Genetically, albinism is classified according to the type of genetic mutation present. There are seven types of non-syndromic OCA identified to date; of these, Type 1 OCA (OCA1) and Type 2 OCA (OCA2) are the most common. OCA1 is considered the most prevalent type globally,^56^ affecting different ethnic groups and characterized by loss of function of the tyrosinase enzyme, as a result of a mutation in the TYR gene. Tyrosine is the critical enzyme in the biosynthesis of the brownish-black eumelanin and yellow pheomelanin. Individuals with OCA1A have non-functional TYR with total absence of melanin production, whereas individuals with OCA1B have some function of tyrosinase activity with limited production of melanin.^57^ OCA2 is the most prevalent form of albinism in Africa.^5^, ^35^, ^53^ The disorder affects those of African descent more often than Caucasians and is characterized by mutation in the OCA2 gene (previously known as the P gene), which encodes the P protein.^5^, ^57^ Its exact functions are not fully understood, but the P protein appears to be involved with the transport of proteins to melanosomes, stabilizing the melanosomal protein complex and the regulation of the pH of the melanosome and/or metabolism of glutathione, all of which are key for melanin production.^5^, ^34^, ^57^, ^58^, ^59^ Albinos with the OCA2 phenotype have no eumelanin, but have some degree of pheomelanin, which can progressively increase with age.^57^, ^58^, ^59^, ^60^

The OCA3 and OCA4 phenotypes of albinism are caused by mutations in genes encoding tyrosinase-related protein 1 (TYRP1) and membrane-associated transporter protein (MATP), respectively.^60^ TYPR1 is an enzyme that stabilizes tyrosinase. Mutations in TYPR1 are associated with the early degradation of tyrosinase and late maturation of melanosomes.^7^ MATP acts as a melanosomal membrane transporter protein necessary for melanin biosynthesis. Mutations in the MATP gene cause hypopigmentation and the OCA4 phenotype of albinism.^7^ The OCA5 phenotype is linked to a specific, as yet unidentified gene, mapped to the region of the 4q24 chromosome, which was discovered in members of a consanguineous Pakistani family.^37^, ^61^

In early 2013, a team of Chinese researchers reported the use of exome sequencing to reveal the molecular basis of albinism in an affected family. They discovered that mutations in SLC24A5, a gene that encodes a solute transporter protein, was associated with a new form of OCA, denominated OCA6. SLC24A5 mutations were detected in patients of different ethnicities, indicating that OCA6 is not confined to the Chinese population. Recent results indicate an important role of SLC24A5 in the maturation of melanosomes, melanosomal architecture, and in proper melanin biosynthesis. Animal studies have revealed that mutation of this gene leads to a reduction in the size and density of melanosomes.^38^, ^39^ Also in 2013, a new gene associated with albinism was discovered among individuals with OCA from the Faroe Islands. The C10orf11 gene was identified using gene mapping of a consanguineous family. In addition, a mutation in the same C10orf11 gene was found in an albino from Lithuania. These data strongly suggest a role of this new gene in the differentiation of melanocytes.^5^, ^21^

At birth, people with different phenotypic forms of OCA present, in general, white hair and very fair or whitish-pink skin. Individuals with OCA1B, OCA2, OCA3, OCA4, OCA5, OCA6, or OCA7 go on to acquire some pigmentation throughout life, but those with OCA1A remain completely depigmented.^37^, ^60^, ^61^

### Melanin and skin cancer

Skin melanin, particularly eumelanin (brown/black), provides protection against solar radiation and oxidative damage to DNA induced by stress, whereby individuals with dark skin have a lower rate of skin cancer than those with fair skin. However, photoprotection conferred by melanin is not total, even in dark-skinned individuals, who also suffer solar radiation-induced DNA damage. This damage generally occurs at a degree that is reversible by the mechanisms of cellular DNA repair, thus reducing the risk of malignant transformation. By contrast, in fair-skinned individuals with insufficient melanin to provide effective protection against solar radiation, the extent of DNA damage can exceed the repair ability of these mechanisms, with major risk of malignant transformation.^62^, ^63^ Albinos who have little or no skin melanin are therefore highly susceptible to UV-induced cancers.

The melanin present in OCA is predominantly pheomelanin (yellow/red), with minimal production of eumelanin.^64^ Eumelanin plays an important photoprotective role. Although pheomelanin offers some photoprotection against solar radiation, carcinogenic reactive oxygen species (ROS) are generated during its biosynthesis. In albinos, a reduction in photoprotection due to the small amount of eumelanin present – as well as an increase in ROS derived from pheomelanin – are implicated in keratinocyte cancers.^60^

The biosynthesis of both types of melanin, brown/black eumelanin and yellow/red pheomelanin, is controlled largely by the melanocortin-1 receptor (MC1R) in melanocytes. Albinos with the OCA2 phenotype who have polymorphic genetic variants of MC1R, in contrast to the majority of other albinos, can have reddish hair and a yellowed skin tone.^65^ The activity of some variants of MC1R can neutralize apoptosis and reduce the DNA repair ability in melanocytes. It can also, indirectly, reduce the protection of keratinocytes against DNA damage induced by solar radiation, due to the reduced production of eumelanin, thereby increasing the risk of skin cancer. The variants of the MC1R gene are associated not only with the dysregulated production of melanin and reduced tanning ability, but also with the modulation of immune inflammatory responses important in immunologic surveillance and destruction of keratinocytes transformed by solar radiation.^49^, ^64^, ^66^ Hence, genetic polymorphisms of other coding genes involved in the biosynthesis of melanin (TYR and TYRP1), besides determining skin pigmentation, are indeed factors contributing to the risk of developing skin cancer.^49^ It has been suggested that the functionally active tyrosinase has the ability to protect against oxidative damage to DNA.^67^ Thus, in summary, lack of melanin and exposure to intense UVR increase the risk of developing skin-cancer.^68^, ^69^, ^70^

Differences in sensitivity to carcinogenesis of the skin can be expected depending on the mutation in OCA. There is no convincing evidence supporting differences in risks across the various types of albinism. The assumption that the presence of low levels of pigment confers some photoprotection, lowering the risk of developing skin cancer in albinos, has been put in doubt.^60^ Reports in the literature suggest that patients with OCA1A have a lower risk of developing skin cancer compared with other types of OCA. This is supported by the fact that the melanin polymer pheomelanin is produced mainly when melanogenesis remains at base level, such as in OCA1B, 2, 3, 4, 5, 6, and 7. Pheomelanin promotes the production of ROS. The damaging effect of ROS on DNA is well known.^71^ Because patients with OCA1A do not synthesize pheomelanin, the risk of producing ROS induced by UVR is lower. Another argument that suggests OCA1A patients may be less vulnerable to skin cancers stems from comparisons with vitiligo. In patients whose white patches have no melanin, a negative correlation between vitiligo and skin cancer has been reported.^72^ Another explanation for this might be that the elevated levels of glutathione peroxidase and superoxide dismutase (SOD) inactivate ROS, conferring protection against oxidative damage and, consequently reducing the risk of skin cancer.^73^ It would be worthwhile examining this in the white skin of patients with OCA1A to distinguish between the susceptibilities to skin cancer induced by UVR; the various mutations that cause OCA should be considered together in studies on single nucleotide polymorphisms (SNPs), showing a significant association with risk of skin cancer.^74^ In conclusion, the absence of pheomelanin and the possible inactivation of ROS by antioxidant enzymes means that the risk of skin cancer induced by UV radiation in patients OCA1A may well be lower than in other types OCA. Studies investigating the differences in ROS concentration should be carried out to provide a valid comparison of the risk of skin cancer between OCA1A and other types of albinism. Additionally, information obtained from SNPs should be taken into account, providing additional data on the molecular mechanisms of skin cancer in albinism.^60^

### Malignant transformation induced by solar radiation in albinism

OCA predisposes to keratinocyte skin cancers, *i.e.*, basal cell carcinoma (BCC) and squamous cell carcinoma (SCC), particularly in more sun-exposed areas.^11^, ^75^ Given that individuals with OCA are typically more prone to sunburn,^5^ the progenitor keratinocytes of basal cells of the skin of albinos exposed to sun are at greater risk of undergoing malignant transformation induced by UV radiation. SCCs in albinos may develop *de novo* or from premalignant actinic lesions, such as actinic keratosis, in which keratinocytes can undergo initial transformation induced by solar radiation. The keratinocytes exhibit different degrees of DNA damage, depending on the intensity and duration of exposure to sunlight. Normally, the suppressor gene of p53 tumors disrupts the cell cycle, allowing the repair of the damaged DNA or promotion of apoptosis if the damage to the DNA is irreparable. However, if the solar radiation induces mutation in the p53 itself, rendering it dysfunctional, there will be propagation of the damaged DNA by cell division, resulting in a pre-cancerous epithelial field composed of a clone of initially transformed keratinocytes with genomic instability. This genomic instability predisposes the initial transformation of the keratinocytes to further genetic alterations and can lead to clonal divergence processes, with consequent expansion of keratinocytes with selective growth advantage, ultimately giving rise to a full-blown SCC.^50^, ^62^, ^76^, ^77^

The risk of skin cancer is proportional to the accumulated amount of UV radiation absorbed by the keratinocytes^78^; the potential for malignant alteration is determined by the number of genetic insults. Thus, a high frequency of shorter exposures to sunlight are more likely to be carcinogenic than less frequent longer exposures,^78^ since each exposure event can potentially cause a change. The more genetic alterations that occur, the greater the chance of malignant transformation.

As albinos are photosensitive and tend to burn easily, the local inflammation induced by the solar radiation in the skin can be an additional factor contributing to increased proliferation and longevity of keratinocytes, favoring malignant transformation. Following sunburn, the ROS derived from local inflammatory cells can directly damage DNA, and/or dysregulate the mechanisms, not only of DNA repair and cell cycle control, but also of apoptosis, thus promoting evolution to skin cancer.^79^

At a molecular level, DNA damage induced by UV radiation is characterized by substitution of specific nucleotides, particularly the substitutions C > T and CC > TT found in the p53 gene that encode the p53 protein, which normally regulate the cell cycle, apoptosis, and DNA repair. Solar radiation regularly provokes these genetic changes, referred to as “signatures” associated with UV exposition; these “signed” mutations lead to malignant transformation from solar radiation causing skin cancer.^78^, ^80^

The initially transformed keratinocytes are immunogenic and produce immune responses that can modulate or control tumorigenesis; however, immunosuppression induced by solar radiation can critically disrupt this protection mechanism.^81^ Therefore, exposure to UV rays is very harmful to hypopigmented skin of albinos. Lack of melanin predisposes this population to severe cutaneous damage. Most of this damage occurs in the body regions most exposed to the sun, such as the face, ears, neck, and shoulders. Skin lesions include sunburns, blisters, solar elastosis/keratosis, ephelides, lentigo, and skin cancer.^24^, ^68^, ^82^, ^83^

## Climatic factors and skin cancer in albinism

### Climatic factors in Brazil

Brazil is transected in the North by the Equator and in the South by the Tropic of Capricorn, thus 92% of its area is within the tropics, providing very high levels of solar radiation. Despite some variations in latitude, altitude, and atmospheric characteristics in different regions, meteorological studies show that UVR tends to be very high throughout the country. This holds true even during the winter season and outside the hours considered critical for solar exposure, typically attaining high levels on the ultraviolet index scale (UIV) published by the World Health Organization (WHO), *i.e*., from very high (UIV 8–10) to extreme (UVI 11+). Daily doses of radiation are, on average, higher in the North and Northeast regions, reaching 20 times the doses recommended by the WHO even in winter, but critical levels of over 15 are also recorded in the Southeast of the country during the summer.^84^, ^85^ Studies have shown that the incidence of squamous cell carcinoma doubles for every eight to ten degree decrease in latitude, peaking at the Equator.^86^ The UV dose per time unit in the Equator is around 200% of the dose in Europe or the Northern United States.^87^ This accumulation of UV radiation occurs more readily in albino skin owing to melanin deficiency, making Brazil extremely unfavorable for this highly vulnerable population.

### Solar radiation, actinic damage, and skin cancer

The prevalence of actinic keratosis depends on skin type, geographic location, and length of exposure to sunlight. Left untreated, these lesions can evolve to SCC.^88^, ^89^ As previously outlined, Brazil has a high UVR index, favoring the emergence of skin cancer in its population with lower phototypes.^90^ A study in Australia conducted by Green and Batistutta^91^ showed that fair skin is the most important risk factor for developing cancer. Maia et al.^92^ classified the skin type of 259 patients with SCC according to Fitzpatrick's criteria.^93^ The fair-skin types I and II, the least pigmented of the six types classified, have greater risk of developing the cancer. These studies indicate a direct relationship between fair-skin and greater occurrence of skin cancer, a group that includes those with albinism.

**Figure 4:**
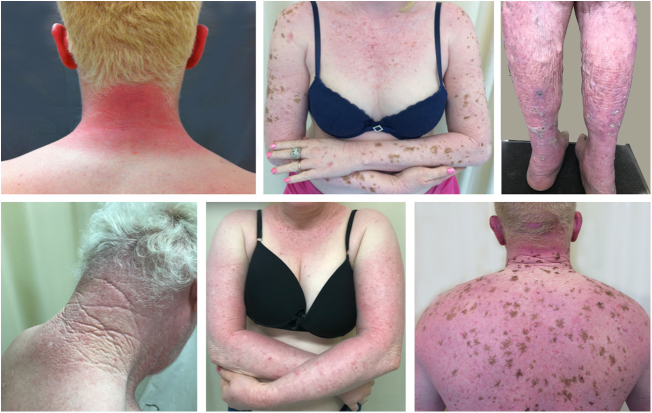
Actinic damage in albinism. Albino patients presenting actinic damage in photoexposed areas.

**Figure 5:**
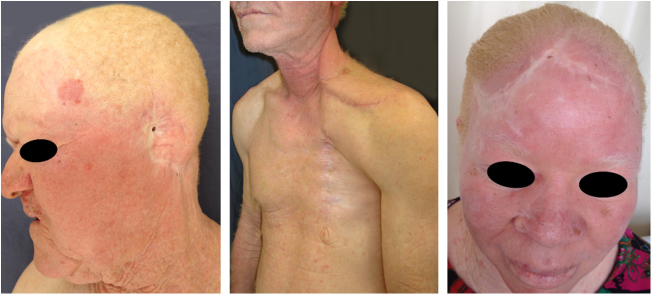
Sequelae of skin cancer. Albino patients with multiple scars and mutilations due to previous surgeries for excision of skin cancers.

Actinic and skin cancers are generally found in middle-aged and elderly fair-skinned individuals living in sunny areas, such as Australia and South Africa.^94^ An estimated 80% of skin cancers are diagnosed in those aged over 55 years.^95^ A recent study performed at the Skin Oncology Sector of the Dermatology Outpatient Clinic of the Santa Casa de Misericórdia de São Paulo (not yet published), revealed that the age of 945 patients with first-time non-melanoma skin cancer (NMSC) averaged 68 years, while 1.7% were under 40 years of age and 90.3% were over 50 years. A similar study (also yet unpublished), composed of 146 patients on a pro-albino care program seen by the same institution, found that the mean age of albino patients with skin cancer was 47 years. As expected, failure to use sun protection and sunburn were significantly associated with cancer occurrence. The population studied also exhibited a high rate of actinic damage (Fig. 4): sunburn (72%), actinic keratosis (45%), elastosis (57%), lentigo (25%), and skin cancer (26%). Unfortunately, these injuries were highly prevalent, with rates similar to those reported in African studies.^27^ These lesions lead to the need for albinos to undergo multiple treatments, surgeries, and even disfigurements, which could be averted through prevention or early intervention (Fig. 5). The minimum ages at which this damage occurred was also found to be precocious, as reported in Africa,^83^ with the lowest age for actinic keratosis being 21 years, solar elastosis observed in a 6-year-old patient, and skin cancer at 23 years of age.

In the negro population with albinism in Africa, actinic damage can be seen in young children. Non-melanoma skin cancer, including basal cell and squamous cell carcinomas, are common in this susceptible group with albinism. Studies in West, East, and South Africa reveal the extent of vulnerability of those affected by albinism.^27^, ^96^ Many studies have documented a high rate of actinic keratosis and skin cancers in these patients,^24^, ^27^, ^68^, ^82^, ^97^ especially when compared to the low prevalence of skin cancer in the normally pigmented negro population from Africa, in which skin cancers are rare.^98^, ^99^, ^100^, ^101^, ^102^, ^103^ The hot climate and farming activities in this community limit the practical use of clothing with effective sun protection; this factor alone can increase the rate of skin cancer^87^, ^88^ Also highlighted in previous reports,^24^, ^27^, ^98^ this relationship can be confirmed by studies of population of albinos in which the frequency of malignant skin abnormalities is much higher in those living in equatorial regions than those living farther from the Equator.

A study by Lookingbill et al. of 164 albinos from rural equatorial Africa (North Tanzania) revealed that, with the exception of four babies, all patients had sunburn. A high rate of actinic keratosis and skin cancer was found in the adults, but actinic skin damage was also observed in young children. Wrinkling on the back of hands and solar elastosis on the back of the neck was present in virtually all patients aged over 10 years. Actinic cheilitis was also present in many children and in 93% of patients over the age of 10. The youngest patient with actinic cheilitis was 8 years old. In patients aged over 20 years, 91% had actinic keratosis, whereas among patients over 30 years, this rate was 100%.^83^

A recent study conducted in Kenya (located along the Equator) involving 151 albinos revealed the presence of serious skin lesions in 80% of patients. Most patients were in the age group of 21–30 years. The frequency of premalignant and malignant lesions was as follows: actinic cheilitis (18%), solar elastosis (12%), actinic keratosis (37%), and skin cancer (13%).^104^ A prospective assessment of 64 French albino patients revealed a medical history of prior skin cancer in three adults (4.6%). Actinic keratosis was detected in 9% of the patients.^42^ Very few patients in this European study reported sunburns, confirming a satisfactory level of sun protection. Similarly, dermatological characterization in Italian albinos involving 200 patients revealed the presence of actinic keratosis in three patients (1.5%) and skin cancer (melanoma) in one.^105^ This finding might be explained by lower sun exposure at European latitudes.

### Skin cancer in albinos

Skin cancer is the most common malignancy among Caucasians. The condition accounts for around 20–30% of all cancers in Caucasians and 1–2% in those with pigmented skin.^101^ Skin cancer is one of the leading causes of morbidity and mortality among albinos who develop premalignant and malignant lesions at a younger age and who have advanced skin cancer by the third and fourth decades of life.^27^, ^27^ Previous studies in Nigeria^97^ and Tanzania^102^ report that only a few albinos survive beyond the age of 30 years. By the third decade of life, many negro albinos in Africa will have developed potentially fatal SCCs,^7^, ^69^ but if diagnosed at an early stage, SCC is curable by surgical excision.

Studies show that SCC risk in albino negroes is 1000 times greater than for the general population, with the head and neck being the most commonly affected areas.^63^, ^69^, ^75^ SCC is more frequent, has a more aggressive course, and tends to have a higher recurrence rate in negro albinos than in normally pigmented individuals.^7^, ^8^, ^103^

A study carried out in Nigeria revealed albinos represented 67% of patients treated for primary skin cancers at a university teaching hospital, 61% of whom were aged under 40 years.^12^ The presence of skin cancer was investigated in 111 albinos from a negro population from Johannesburg, South Africa. The overall rate was 23%, with increased risk with age.^68^ In a review involving 1000 Nigerian albinos, Okoro found none over the age of 20 free of pre-malignant or malignant skin lesions induced by solar radiation.^27^ A similar finding was also reported by Luande et al. in their review of 350 albinos in Dar-es-Salam. In the cited study, the peak age of patients with advanced skin cancer was the fourth decade of life.^24^ Another study was carried out in Tanzania involving a histological review of 134 biopsies from 86 albino patients diagnosed with skin cancer seen at a dermatologic center in Tanzania. The age of the youngest patient was 18 and the oldest 68 years, while the mean age was 35 years.^88^

In Brazil, there are scant studies with clinical data on albinism, and the only two such studies available were conducted in Bahia. One was published in 2007 and involved 40 albinos affiliated with the APALPA. The study showed that 42% of the albinos had skin lesions and 47% did not use sunscreen regularly. A substantial proportion of the albinos who developed skin lesions did not use sunscreens or seldom used them.^44^ A retrospective study was published in 2013 of data collected from 22 albinos and 30 non-albinos (24–89 years) with cancerous lesions and 24 albinos without skin lesions. The mean age of the albinos with cancers was 34 years *vs.* 65 years for non-albinos. Using the Yates test, a significant relationship between use of sunscreen and absence of development of skin tumors was determined. Among those albinos who had used sunscreens since childhood, only two (11%) had developed skin cancer.^48^

The prevalence of skin cancer in patients studied from the Pro-Albino Program (Brazil, *n* = 146) (26%) was similar to that found in albinos by researchers in Tanzania (25%),^83^ South Africa (23%),^68^ and much higher than the rates found in Europe of 6% in France and of 0.5% in Italy.^42^, ^105^ Therefore, comparison of the present results with data in the literature reveals a great similarity with reports of studies in Africa, but a major difference with the findings of European groups. This illustrates the major influence of environmental factors and socioeconomic and cultural conditions on the morbimortality associated with albinism. Special focus and approaches will be needed when considering preventive measures. Those data make clear the fundamental importance of effective guidance and attitudes regarding sun protection in regions with a high incidence of solar radiation.

### Types of skin cancer in albinism

NMSC accounts for 90% of all skin cancers. The most common skin tumors in Brazil and globally are BCC (70–80% of diagnosed cases) and SCC (20–25% of cases). BCC is three to four times more common than SCC in the white population. Both have high cure rates if detected early and only a small proportion of cases are fatal. These cancers have the highest incidence and lowest lethality of all the skin cancers.^89^, ^106^, ^107^, ^108^, ^109^

A retrospective study involving a review of medical records of 945 patients with first-time NMSC conducted at the Santa Casa de São Paulo found that 82% had a diagnosis of BCC and 18% had SCC. The body sites most affected were the head and neck (81% of cases), followed by the trunk (11%) and the limbs (8%; data not published). By contrast, SCC was the most common cutaneous malignancy observed in African studies.^11^, ^12^, ^27^, ^68^, ^69^, ^97^, ^110^ A study of African albinos reported a SCC to BCC prevalence ratio of 1.2:1. The head and neck were the sites most commonly affected by the skin cancer (56%).^88^ Previous studies have also reported a predominance of SCC in albinos, with SCCs occurring at rates three to six times higher than BCCs.^11^, ^12^, ^68^, ^97^, ^111^ A review of 775 normally-pigmented Africans and 18 albino Africans with malignant tumors showed that SCC was the most common tumor type, with the head and neck regions most affected.^97^ In another study carried out in South Africa of 111 negro albinos, the head was the most commonly affected site and SCC was more frequent than basal cell carcinoma. No melanoma cases were detected.^68^

**Figure 6:**
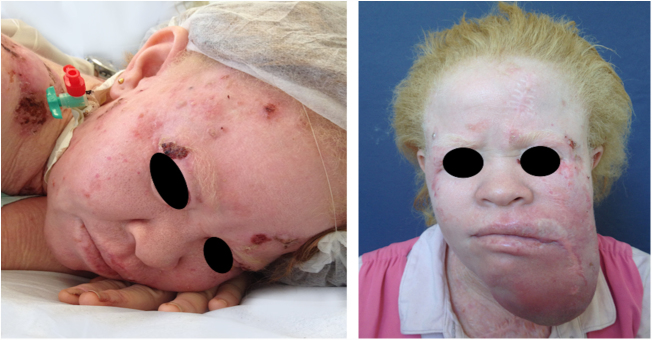
Morbimortality associated with skin cancer. Young albino patient, presenting multiple ulcerated tumors. He died at age 27 due to metastatic squamous cell carcinoma.

**Figure 7:**
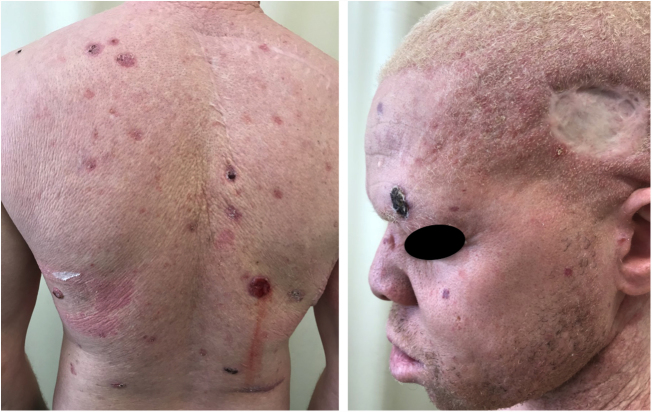
Morbimortality associated with skin cancer. Young albino patient with multiple tumors (BCC and SCC) and surgical scars from previous excisions.

**Figure 8:**
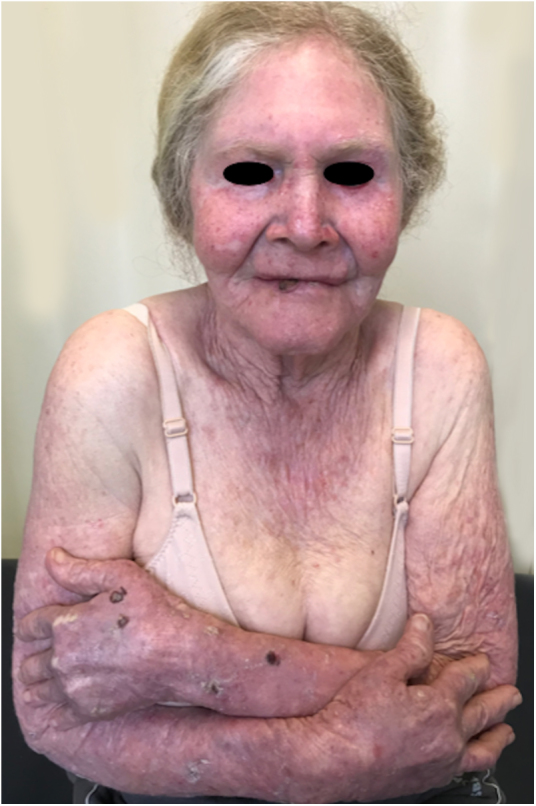
Cumulative solar damage – unprotected exposure. Elderly albino patient with multiple actinic damage in photoexposed areas. History of basal cell carcinoma and squamous cell carcinoma.

In the cited study of patients in the Pro-Albino Program, of the 37 (26% of sample; *n* = 146) patients with previous or current history of skin cancer, the histological type was identified in 29, comprising 62% BCC, 51% SCC, and 7% melanoma, at a BCC/SCC ratio of 1.2:1. This proportion differs slightly to that reported for the non-albino population, albeit with BCC still predominating over SCC cases. Of these patients, 14% had both carcinoma types (BCC and SCC). Some patients had multiple ulcerated tumors (Figure 6, Figure 7, Figure 8). The site of tumors was preferentially the head and neck (43%), followed by the trunk (37%) and limbs (20%), mirroring the tendency of greater prevalence in areas most exposed to the sun.^11^, ^12^, ^24^, ^68^, ^83^, ^96^, ^97^, ^104^, ^110^ Reporting similar results to those of the present study, another investigation carried out in Bahia, Brazil showed that of the albinos with skin cancer (*n* = 22), 56% had basal cell carcinoma.^48^

The relatively high predominance of SCCs over BCCs in the African studies may have been due to small sample sizes, four of which comprised fewer than 20 patients. The majority of these studies were also based on excisional biopsies of advanced tumors. The small slow-growing BCC may have been missed in earlier studies. Biopsies were taken from patients submitted to routine dermatological exams in only one of the studies, which showed an essentially balanced rate of SCC and BCC (1.2:1),^88^ as seen in the present study, in which the proportion of SCC and BCC was inverted (1:1.2), but very similar from a quantitative standpoint. Based on this information, it has been suggested that non-melanoma skin cancers are predominant in albinos and that the proportion of SCCs and BCCs is almost identical.

In Africa, negro albinos are often subjected to discrimination due to superstitious beliefs and stigma associated with the condition.^62^ Albinos are often shunned by their communities, with consequent delays in seeking and receiving medical treatment. Thus, by the time the condition is diagnosed, the SCCs in African albinos are often already advanced.^12^, ^62^ Reports indicate that, on average, negro albinos in Africa seek treatment 14–26 months after the onset of actinic lesions.^12^, ^69^, ^88^, ^97^, ^111^ Unfortunately, around 40% of these albinos with skin cancer do not conclude treatment due to financial problems^11^ or miss follow-up sessions because of distance from medical facilities. These reasons might explain why SCCs are more frequent and tend to have greater recurrence rates in negro albinos in Africa than in normally-pigmented individuals.^7^, ^8^, ^103^

**Figure 9:**
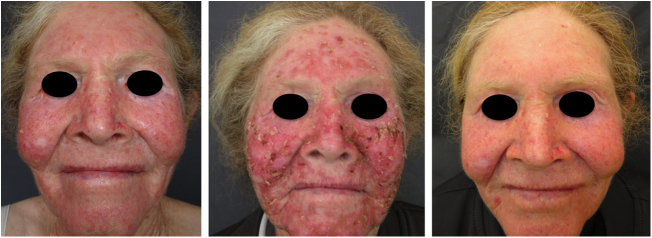
Treatment of the cancerization field. Albino patient with multiple actinic keratoses on the face. The image shows the result of treatment of the cancerization field with 5-fluorouracil.

**Figure 10:**
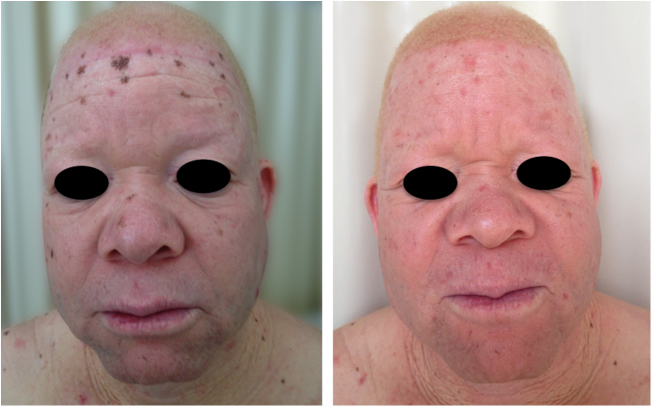
Treatment of actinic damage. Albino patient with multiple actinic keratoses and solar lentigines treated with cryotherapy.

A recent study in Kenya found that BCC was more common than SCC in albinos.^104^ The lower SCC rates were attributed to early treatment of actinic keratoses, preventing their transformation into malignant SCCs. There is no known correlation between actinic keratosis and BCC. These findings highlight the importance of early diagnosis and treatment of premalignant lesions, which could be easily achieved *via* care programs aimed at treating this population. The treatments of choice under the authors’ Pro-Albino Program for premalignant lesions (actinic keratoses) are cryotherapy and topical chemotherapy with 5-fluorouracil (Figure 9, Figure 10), *i.e.*, relatively simple, effective, low-cost interventions.

## Alternatives for minimizing skin cancer risk in albinos

The goals of skin cancer management in public health are to reduce incidence and provide early detection and immediate treatment of the disease when it occurs. Universal precautions against sun exposure should be introduced during early childhood, continue through life, and should include reducing outdoor activities during peak sunlight hours (10–16 h), the use of protective clothing to cover as much skin as possible, wide brim hats, sunglasses, and sunscreen for exposed skin and lips.^62^, ^76^, ^112^ Based on these considerations, the crucial role of the dermatologist is clear in the management of this condition and particularly in screening to detect early skin cancers.

Studies in Africa have shown that the prevalence of skin cancers could be reduced in albinos^12^ if health teams were regularly deployed to remote villages to screen for malignant and premalignant lesions in the albino population and to educate them on the harmful effects of exposure to solar radiation. This would need to be complemented by medical centers, where early treatment of the cancer could be provided,^26^ and by establishing educational support groups. Other studies assessing the impact of albino care programs in Africa have shown an increase in life expectancy as a result of greater awareness and early treatment of skin cancer.^83^, ^113^

The sustained use of sunscreen and other photoprotective measures is pivotal in the preventive management of actinic damage and of skin cancer in albinos.^76^, ^112^ The use of sunscreen from childhood reduces the chances of developing skin cancer in the general population by around 78%,^114^ a figure likely even greater in albinos. In practice, there is major difficulty in acquiring these products due to a total lack of access and resources. It is of vital importance that the government provides these products free of charge to the albino population, because this would reduce the secondary costs of treating skin cancer, loss of work productivity, and the morbimortality associated with the condition.

Health communication has also proven effective in reducing the morbimortality associated with albinism.^26^, ^102^, ^113^ Pertinent information on preventive measures should be given at each visit. Organizing meetings with patients can also be useful, in which these issues and other problems related to albinism can be raised and discussed. Communication in schools and specific guidance on professional pursuits in a bid to minimize sun exposure are important. The present authors advocate an interactive social learning approach for extension programs, support groups, and workshops focused on the management of albinism.

Estimates of albinism prevalence suggest that there are a significant number of individuals living with albinism in Brazil. The evidence shows that a large proportion of these individuals live in poor socioeconomic conditions and require social and healthcare services that are lacking. This scenario reiterates the need for greater awareness and public health intervention for albinism.

## Psychological and social issues: stigma, prejudice, and discrimination

Besides concerns over physical health, individuals with albinism also have to cope with psychological and social challenges. In Nigeria, a study collected reports from albinos, showing that they avoided social situations so as not to be noticed. They were more emotionally unstable and had less assertive personalities than people without albinism. In addition, they deemed society as insensitive and felt rejected, even when they had close friends.^112^ Most social discrimination in Africa appears to stem from a lack of education among communities about the causes of albinism. There is limited awareness of its genetic inheritance, giving rise to numerous deep-rooted myths and superstitions.^22^ For example, some beliefs link albinism to conception during menstruation (culturally unacceptable) or consider albinism a punishment by the gods for mistakes made by an ancestor.^27^ This socially-entrenched discrimination can negatively impact the quality of life of people with albinism. For example, this group is more likely to drop out of school and encounter difficulties in employment and marriage compared to the population at large. Moreover, the families of albinos may also be subjected to discrimination by the community. As a result of traditional myths surrounding the cause of albinism, mothers of affected offspring may be subjected to major stigma and psychological distress.^22^, ^26^, ^28^, ^102^, ^115^, ^116^ In Brazil, owing to total ignorance of the condition, this distress involves, for instance, questions regarding paternity at the time of birth of an albino infant within a family of African or mixed descent, whereby the mother and baby suffer prejudice from within the family and the general community.

Individuals with albinism also face social discrimination as a result of their different appearance. Studies show that most countries report lack of knowledge by the general public. The studies also reveal that many people with albinism do not fully understand their condition. This social discrimination is viewed as a barrier to building relationships and to finding and holding down a job. Therefore, most people with albinism generally have lower economic status in their society. In light of these difficulties, it is unsurprising that many studies have reported abuse and psychological problems in this population.^26^ No specific studies on these factors are available for Brazil; however, the experiences acquired from the Pro-Albino Program and from numerous personal accounts indicate that Brazilian albinos face similar problems. One of the Program's goals is to address these issues in order to eliminate stigma and prejudice, and to promote social inclusion.

Albinism is a disorder that affects individuals and their families from a medical, social, and psychological perspective. For many, the social and psychological aspects can be a greater burden than the medical issues. Although small compared with other major public health problems, the number of individuals affected by the condition – and the even greater number indirectly impacted – make albinism a public health problem that warrants greater attention, particularly to increase awareness and knowledge about the condition.

## Diagnosis

Albinism is diagnosed based on clinical observations and molecular genetic analysis considering the following factors:

• Full physical exam, including checking pigmentation of the skin, hair, and eyes^4^, ^5^;
• Thorough ocular exam, including assessment of possible nystagmus, strabismus, refractive deficits, photophobia, and iris transillumination. A visual inspection of the retina is also carried out to determine whether there are signs of abnormal development^117^;
• Comparison of pigmentation of the albino patient against that of other family members;
• Review of family and personal history, including the existence of prolonged bleeding, excessive bruising, intestinal, pulmonary, or neurological abnormalities, or repeated infections.

### Differential diagnosis

Based on hair and cutaneous findings (hypopigmentation):

• OCA syndromes;
• Hermansky-Pudlak syndrome;
• Chediak-Higashi syndrome;
• Angelman syndrome and Prader-Willi syndrome;
• Vici syndrome: autosomal recessive disorder characterized by agenesis of the corpus collosum; hypopigmentation of the hair and skin, microcephaly, immunodeficiency, heart defects, growth deficit, cataracts, cleft lip/palate, and neurological abnormalities;
• Waardenburg syndrome type II: Mutation of the autosomal dominant MITF gene, characterized by heterogeneous hypopigmentation of the skin, white locks or prematurely gray hair, heterochromia of the iris and sensorineural hearing loss;
• Albinism-deafness of Tietz syndrome: mutation of the autosomal dominant MITF gene, characterized by white eyebrows and eyelashes, iris hypopigmentation, normal visual acuity, and sensorineural hearing loss;
• Griscelli syndrome: autosomal recessive mutation in myosin, together with its receptors and ligands; melanocytes do not transfer melanosomes to dendrites and peripheral keratinocytes, leading to attenuation of skin and hair color; presents with hypopigmentation, silvery-gray hair, immunodeficiency, reduced visual acuity with abnormal ocular movements, pancytopenia, hemophagocytic syndrome, and cerebral demyelination.^118^

### Genetic diagnosis

The type of albinism and genetic inheritance can be determined through molecular genetic diagnosis. Genetic analysis also allows proper genetic counseling and early diagnosis of syndromic forms (Hermansky-Pudlak and Chediak-Higashi), which can present initially as non-syndromic forms and develop serious complications at a later age. These complications can be prevented with guidance and early therapeutic interventions.^4^, ^5^, ^119^, ^120^

Genetic counseling prior to the fertile years is beneficial to parents of albino children considering having further children, as well as for the albinism patient and their siblings. Albinism is an obligate homozygote condition with 100% chance of transmission of the defective gene. Coordinated genetic testing of the unaffected partner is possible if the pathogenic variant is known. This will confirm that descendants have the potential to inherit the condition, if the partner is a carrier of the same pathogenic variant or only obligate carriers if the partner only has wild type genes. A couple who already has an albino child will have a 25% chance of having another child with albinism, a 50% chance of having children who are carriers of the gene, and 25% of having non-carrier offspring. Therefore, it is assumed that if one of the parents is non-albino, the chance of having a second albino child is 50% after confirmed albino descendancy. It is important to advise that non-albino siblings have a 67% chance of being carriers before they consider having children. Also, if both parents carry genes for different types of albinism, no child will be born with albinism, but the children run the risk of being heterozygous for both mutant alleles.^118^

In summary, genetic diagnosis is of fundamental importance in the management of albino patients. Molecular study enables classification of subtypes (not possible by phenotypic clinical assessment alone), diagnosis of non-clinically evident cases, provision of genetic counseling, and identification of syndromic forms.

## Treatment

Given that the condition is a genetic disorder, albinism has no cure. Treatment centers on administering proper ophthalmologic care and on skin monitoring for signs of abnormalities and prevention of sun damage. Treatment generally encompasses the following:

• Eye care: ophthalmologic assessment in the first months of life, regular ophthalmologic exam, corrective lenses, ocular physiotherapy, surgical interventions when necessary, guidance for learning – learning aids and special consideration in the classroom (high-contrast reading material, printed texts and spreadsheets, large scale display settings on computers, among others). This approach helps overcome the learning disabilities associated with visual deficits^117^, ^118^, ^121^;
• Skin care and prevention of skin cancer: guidance on actinic damage prevention and regular clinical and cutaneous dermatoscopy assessment to detect skin cancer or precursor lesions. Interventions such as application of liquid nitrogen, topical chemotherapy, curettage, electrocauterization, and surgery are performed when needed.

There is no substitute for sun protection throughout the lifespan in albinism and its importance cannot be underestimated. Individuals should be informed about avoidance of prolonged exposure to UV light, direct exposure in critical hours of high incidence of radiation (10:00–15:00), and avoidance of medications that can increase photosensitivity. Any outdoor activity, however short, should be preceded by applying sunscreen (SPF 30+) with copious frequent reapplication (every two hours) when in the sun. Additional protective measures should be taken, such as the use of protective clothing, hats, and sunglasses. Self-examination and self-education about the criteria for skin cancer and immediate referral to a dermatologist in the event of any suspect lesion must be emphasized. Monitoring with a dermatologist should commence early (childhood) to explain the benefits and options regarding sun protection.^11^, ^104^, ^118^

Syndromic forms: patients with the Hermansky-Pudlak or Chediak-Higashi syndromes generally require regular specialized care to meet medical needs and prevent complications.^5^, ^21^, ^118^

Direct therapeutic modalities (in process of investigation and ratification): Nitisinone promotes the accumulation of tyrosine in blood and rat models, suggesting that it may improve pigmentation in OCA1B patients, but a clinical trial is still underway. Aminoglycosides are a potential unconfirmed therapy. Despite reports, L-DOPA led to no improvement in vision in a study of 45 patients. Adeno-associated virus (AAV) vectors are a potential gene therapy that introduces a functional copy of the gene tyrosinase in OCA1 and OA1 patients.^122^, ^123^

## Prognosis

The life expectancy of the population with non-syndromic OCA is similar to that of the general population. There is an increased risk of mortality due to skin cancer. This risk changes based on the amount of relative sun exposure in a given geographic area and certain socioeconomic problems. The socioeconomic issues include restricted access to sunscreen; limited education about sun protection measures; cultural differences in clothing; limited access to health professionals for monitoring, leading to late presentation and delayed treatment; as well as inability to comply with or conclude a course of treatment.

In regions with socioeconomic problems, there is often a palpable stigma associated with albinism, and individuals with the genetic disorder can be victims of persecution, prejudice, violence, and social exclusion.

Albinos have normal intelligence compared to the general population. There is some delay in visual maturation, and this can lead to learning disabilities if the ocular issues are not addressed in a timely manner. In addition, low self-esteem and social alienation can lead to feelings of isolation and depression. Albinos have a high rate of attention deficit disorder.^11^, ^118^, ^121^

## Final considerations

Measures to prevent and control skin cancer in albinos should include the implementation of medical screening programs to identify potentially malignant actinic skin lesions and allow early detection of cancer, and also to make available effective and immediate psychological and dermatological treatment measures.^63^ Although skin cancer is the most common cause of early death in albinos, affected patients can have a normal life expectancy with the provision of adequate skin care.^7^

Irrespective of the geographic location, patients with albinism can feel stigmatized and isolated. Initiatives to raise awareness of albinism are extremely important in all places worldwide. The quality of life of patients can be markedly improved with access to health, support, and adequate guidance. Care with regard to oculocutaneous albinism seeks to reduce morbimortality due to advanced skin cancer and ocular diseases. This is possible through primary dermatologic and ophthalmologic prevention and secondary prevention measures. Through the provision of adequate care for albino patients, the disease course can be changed, and the comorbidities can be treated when present. Additionally, the disease and its distribution in Brazil can be better understood, thereby promoting benefits for the patient and the family members, who often lack adequate information and have no knowledge about the condition. This information can also help eliminate the stigma and prejudice associated with the disease.

## Financial support

None declared.

## Authors's contribution

Carolina Reato Marçon: Statistical analysis; approval of the final version of the manuscript; conceptualization and planning of the study; composition of the manuscript; procurement, analysis, and interpretation of data; intellectual participation in the propedeutic and/or therapeutic conduct in the studied cases; critical review of the literature; critical review of the manuscript.

Marcus Antonio Maia de Olivas Ferreira: Guidance of the study, intellectual participation in the propedeutic and/or therapeutic conduct in the cases studied, critical review of the manuscript.

## Conflicts of interest

None declared.

## Questions

1. The main cause of morbimortality for albinos living in geographic regions with a high solar radiation index is:
  a) Melanoma
  b) Low visual acuity
  c) Keratinocyte cancer
  d) Systemic complications
2. The main syndromic forms of albinism are:
  a) Waardenburg and Prader-Willi
  b) Hermansky-Pudlak and Chediak-Higashi
  c) Chediak-Higashi and Angelman
  d) Hermansky-Pudlak and Waardenburg
3. There are 19 genes related to the different clinical presentations of albinism, including seven for oculocutaneous albinism. The genes responsible for the four main types of non-syndromic albinism – OCA1, OCA2, OCA3, OCA4, are respectively:
  a) TYR, OCA2, TYRP1, and SLC45A2
  b) TYRP1, OCA2, SLC24A5, and C10orf1
  c) TYR, OCA2, SLC45A2, and C10orf1
  d) TYR, OCA2, TYRP1, and SLC24A5
4. The epidemiologic data currently available show that the incidence of albinism is probably highest on the following continent:
  a) South America
  b) Asia
  c) Africa (correct)
  d) North America
5. With regard to the most prevalent genetic types of oculocutaneous albinism (OCA) globally, we may consider:
  a) OCA1 and OCA3
  b) OCA3 and OCA4
  c) OCA2 and OCA4
  d) OCA1 and OCA2
6. Melanocytes have an ectodermic origin, in the neural crest, evolving with cutaneous or extracutaneous migration. Some genes control the proliferation and differentiation of cells from the neural crest and regulate the migration of precursor melanocytes to their final positions. The sites of extracutaneous migration of melanocytes and the master gene regulating development, function and survival of melanocytes, are respectively:
  a) Eyes, leptomeninges, pleura, and MC1R.
  b) Eyes, leptomeninges, hypothalamus, and MITF
  c) Eyes, cochlea, leptomeninges, and MITF
  d) Eyes, adrenal, leptomeninges, and SCF/KIT
7. Regarding the physiology of skin cancer in albinism, the factors directly involved and correlated with gene mutation and its interaction with ultraviolet radiation are:
  a) Reduced quantity of melanin, proportional decrease or absence of eumelanin, p53 dysfunction, and oxidative stress
  b) Reduced quantity of melanin, proportional decrease or absence of pheomelanin, oxidative stress, and immunosuppression
  c) Reduced quantity of melanin, proportional increase of eumelanin, inflammation, and disruption of the cell cycle/apoptosis
  d) Reduced quantity of melanin, proportional increase in pheomelanin, p53 hyperfunction, and ineffectiveness of DNA repair mechanisms
8. The most common actinic damage reported in Brazilian albinos is:
  a) Actinic keratoses, lentigo, and squamous cell carcinoma
  b) Actinic keratoses, solar elastosis, and basal cell carcinoma
  c) Solar elastosis, squamous cell carcinoma, and melanoma
  d) Solar elastosis, actinic keratoses, and melanoma
9. Risk factors currently considered for skin cancer in albinos:
  a) Sun exposure without protection, history of sunburn, and age
  b) Sun exposure without protection, tonality of the hair, and age
  c) History of sunburn, color of the iris, and ethnicity
  d) Genetic type, sunburn, and age
10. Concerning genetic counseling, the chances of a non-albino couple with an albino child having another child with albinism and the chances of their children being carriers of the gene are, respectively:
  a) 50% and 50%
  b) 50% and 67%
  c) 75% and 50%
  d) 25% and 50%

**Table:** 

**Answers**: Paraneoplastic pemphigus: a clinical, laboratorial, and therapeutic overview. An Bras Dermatol. 2019;94(4):388–98.				
1. c	3. b	5. d	7. a	9. b
2. d	4. b	6. a	8. d	10. d
